# Emotional Influences on Eating Behavior and Hunger Awareness Among Generation Z University Students in Greece

**DOI:** 10.3390/nu18101500

**Published:** 2026-05-08

**Authors:** Maria P. Koliou, Chrysoula Karaiskou, Charalampos Eleftheriadis, Achilleas Kontogeorgos, Dimitris Skalkos

**Affiliations:** 1 Laboratory of Food Chemistry, Department of Chemistry, University of Ioannina, 45110 Ioannina, Greece; m.koliou@uoi.gr; 2 Sidroco Holdings Ltd., Nicosia 1082, Cyprus; ckaraiskou@sidroco.com (C.K.); celeftheriadis@sidroco.com (C.E.); 3 Department of Agriculture, International University of Greece, 57001 Thessaloniki, Greece; akontoge@ihu.gr

**Keywords:** emotional eating, Generation Z, eating behavior, emotional hunger, emotional regulation, emotional awareness, overeating, undereating, food enjoyment, behavioral nutrition

## Abstract

Background: Emotional determinants of eating behavior are increasingly recognized as critical components of behavioral nutrition, particularly among Generation Z, a population characterized by heightened emotional reactivity and rapidly shifting dietary patterns. Objectives: This cross-sectional study examined the multidimensional structure of emotional influences on eating behavior among 411 university students in Greece and explored which emotional mechanisms are most closely associated with emotional hunger awareness. Methods: Using the Emotional Influence on Eating Behavior Questionnaire (EIEBQ), four constructs were assessed. Exploratory Factor Analysis supported a refined four-factor structure explaining 63.23% of variance, following the removal of one low-communality item. Results: Participants reported moderate emotional undereating (M = 3.17) and reduced enjoyment of food under emotional distress (M = 3.38), lower reliance on food for emotional overeating (M = 2.86), and high emotional awareness (M = 3.76). Regression analyses, although explaining a small proportion of variance (R^2^ = 0.042), indicated that emotional undereating (β = 0.155, *p* = 0.017) and emotional overeating (β = 0.135, *p* = 0.027) were the most consistent predictors of emotional hunger awareness. Conclusions: These findings suggest that emotional responses—rather than cognitive appraisal—may play a meaningful role in distinguishing emotional from physical hunger. By providing an initial psychometric evaluation and a behavior-focused framework, this study offers preliminary insights into emotional eating mechanisms among Generation Z university students and contributes to the development of targeted interventions promoting healthier and more sustainable dietary behaviors.

## 1. Introduction

Emotions play a central role in shaping eating behavior, influencing not only food choices but also appetite regulation, sensory engagement, and overall dietary patterns. A growing body of research shows that eating is closely intertwined with affective states, psychological stress, and emotion-regulation processes, particularly among adolescents and young adults [1,2,3]. These associations have become increasingly salient in the post-pandemic period, as younger populations experienced heightened emotional volatility, mental-health strain, and disruptions in daily routines. Evidence from recent systematic reviews indicates that these psychosocial changes were accompanied by notable shifts in eating patterns, including increases in both overeating and, in some cases, undereating behaviors among adolescents and young adults [4,5,6].

Generation Z represents a population of particular interest in behavioral nutrition. As digital-native young adults navigating a socially complex and emotionally demanding environment, they report elevated levels of stress, emotional sensitivity, and psychological strain, all of which may influence their eating patterns [7,8,9,10,11]. Understanding how emotions shape food intake, food enjoyment, and self-regulation in this demographic is essential for informing public health strategies and promoting healthier dietary trajectories, given the well-established links between emotion-regulation processes and eating behavior [12,13].

It is important to distinguish between the psychological constructs relevant to emotional eating. Emotional eating refers to changes in food intake triggered by affective states; emotion regulation describes the strategies individuals use to manage or modulate these emotional states [14]; and interoceptive or emotional awareness concerns the ability to recognize internal cues and differentiate emotional from physiological hunger [15,16]. Although related, these mechanisms represent distinct processes that may influence eating behavior in different ways.

Emotional eating itself is not a unidimensional construct. Prior research highlights several distinct yet interrelated mechanisms, including emotional undereating, reduced enjoyment of food under emotional distress, emotional overeating as a coping strategy, and awareness of emotional influences on eating [17,18,19,20,21]. Each mechanism reflects distinct psychological pathways that may either support or undermine adaptive eating behavior. Yet only a limited number of studies have examined these emotional processes within an integrated framework, and research focusing on non-clinical Generation Z samples remains particularly scarce [22,23].

The present study addresses this gap by examining four emotional dimensions of eating behavior among university students in Greece using the Emotional Influence on Eating Behavior Questionnaire (EIEBQ). By exploring emotional undereating, reduced enjoyment of food, emotional overeating, and emotional awareness within the same analytic model, the study aims to provide a clearer understanding of how emotional mechanisms relate to emotional hunger awareness in contemporary young adults.

## 2. Materials and Methods

### 2.1. Study Design and Participants

This cross-sectional study investigated the emotional determinants of eating behavior among young adults. Data collection took place in February 2026, and targeted undergraduate and postgraduate students enrolled at the University of Ioannina (Greece).

A total of 411 students completed the survey. The sample consisted predominantly of female participants (76.6%), with males representing 23.1%. Participants ranged in age from 16 to 28 years, with the majority belonging to the 19–22 age group (63.3%). Most respondents were full-time students (79.8%), while 20.2% combined studies with part-time employment.

Participation was voluntary and anonymous. Students were informed about the purpose of the study and provided electronic consent prior to completing the questionnaire. No incentives were offered. Inclusion criteria required participants to be currently enrolled in higher education and able to complete the questionnaire in Greek or English. The study was conducted in accordance with the Declaration of Helsinki and approved by the institutional ethics committee.

### 2.2. Instrument: Emotional Influence on Eating Behavior Questionnaire (EIEBQ)

Emotional aspects of eating behavior were assessed using the Emotional Influence on Eating Behavior Questionnaire (EIEBQ), a structured self-report instrument developed as part of a broader research program on post-pandemic eating behavior. The EIEBQ was designed as a derived and expanded instrument, based on the ‘Enjoyment of Food’ subscale of the Eating Behavior Questionnaire (EBQ), previously developed by the authors in their earlier work [24]. The development of the EIEBQ builds directly on our previous research program on post-pandemic eating behavior and Generation Z appetitive traits, integrating insights from both our systematic review and empirical clustering study. The EBQ included eight internationally recognized appetitive traits, one of which—*Enjoyment of Food*—captured the emotional modulation of food pleasure and intake.

The development of the EIEBQ followed a theory-driven, construct-based approach, grounded in established models of emotional eating, hedonic coping, and sensory disengagement. Although no formal expert panel or pilot testing was conducted, content validity was ensured through iterative refinement, guided by validated instruments widely used in the field:

- the Dutch Eating Behavior Questionnaire (DEBQ) [25];
- the Emotional Eating Scale (EES) [26];
- the Three-Factor Eating Questionnaire (TFEQ) [27];
- and the Adult Eating Behavior Questionnaire (AEBQ) [28].

These tools assess emotional, hedonic, regulatory, and appetitive dimensions of eating behavior and provide the conceptual foundation for item development.

The EIEBQ comprises 20 items organized into four conceptual domains:

1. Emotional Undereating—Reduced food intake under negative emotional states (irritation, worry, anxiety, sadness, anger).
2. Reduced Enjoyment of Food under Emotional Distress—Diminished sensory engagement and reduced pleasure in eating during emotionally charged situations.
3. Emotional Eating/Emotional Overeating—Use of food as a coping mechanism for stress, tension, sadness, or negative thoughts.
4. Emotional Awareness and Control in Eating—Recognition of emotional triggers, differentiation between emotional and physical hunger, and regulation of eating behavior.

Each domain includes five items. Responses were recorded on a 5-point Likert scale (1 = “Strongly Disagree” to 5 = “Strongly Agree”), with higher scores indicating stronger endorsement of the emotional influence described.

Consistent with established emotional eating instruments (e.g., DEBQ, EES, TFEQ, AEBQ), no definitions or explanatory descriptions of emotional terms (e.g., irritation, worry, anxiety, sadness, anger) were provided to participants. Emotional states are inherently subjective experiences, and providing standardized definitions may artificially constrain or bias self-reported responses. Allowing participants to interpret these terms according to their own emotional perception aligns with best practices in affective and behavioral research. The differentiation among these emotional states was evaluated empirically through the factor-analytic structure of the EIEBQ, which confirmed that participants meaningfully distinguished between the emotional constructs represented in the questionnaire. The present study provides an initial exploratory examination of the EIEBQ’s factor structure. These analyses are not intended as a full validation but as a first step toward establishing the instrument’s psychometric properties. A five-point Likert response format was selected because it is widely used in validated emotional-eating instruments (e.g., DEBQ, EES, TFEQ, AEBQ), offers optimal balance between sensitivity and respondent burden, and provides sufficient variability for reliable factor-analytic and regression-based analyses. Although the EIEBQ has not been previously validated in the Greek population, its development was grounded in internationally established emotional-eating frameworks, and the present study provides the first empirical evidence supporting its structure and applicability in this cultural context.

It is important to note that the development of the EIEBQ followed a theory-driven, construct-based approach rather than a formal content-validation procedure. No expert panel, pilot testing, or cognitive interviewing was conducted; therefore, content validity should be considered preliminary. The instrument was conceptually derived from the ‘Enjoyment of Food’ subscale of the Eating Behavior Questionnaire (EBQ), which has been used in previous research, but the parent scale’s psychometric properties were not re-evaluated in the present study. Accordingly, the EIEBQ is treated as an exploratory instrument whose structure is examined for the first time in this sample.

### 2.3. Procedure

The questionnaire was administered electronically through a secure online platform. Participants accessed the survey via a link distributed through university communication channels and student networks. Completion time was approximately 8–10 min. The survey included demographic questions followed by the 20 EIEBQ items presented in fixed order. Data was screened for completeness prior to analysis. Cases with substantial missing responses were excluded, while isolated missing values were retained and handled using pairwise availability.

### 2.4. Data Handling

Analyses were conducted using all available responses per item. Minor variations in the number of observations across items (408–410) resulted from occasional missing values. Cases with substantial missing data were excluded, whereas isolated missing responses were retained and handled using pairwise availability.

Pairwise deletion was selected because the proportion of missing data was extremely low (0.2–0.7% per item), and preliminary checks indicated that missingness was random and not associated with key demographic or behavioral variables. Although more advanced methods such as multiple imputation or full information maximum likelihood can reduce bias in cases of substantial or systematic missingness, the minimal and random nature of missing data in this dataset made pairwise deletion an acceptable approach for exploratory analyses. Sensitivity checks confirmed that the small variation in *N* (408–411) did not materially affect factor loadings or regression estimates.

### 2.5. Ethical Considerations

The study adhered to the ethical standards of the University of Ioannina and followed the principles of the Declaration of Helsinki. Participation was voluntary, informed consent was obtained electronically, and all responses were collected anonymously. No personal identifiers were recorded.

### 2.6. Statistical Analysis

Descriptive statistics (means, standard deviations, and frequency distributions) were calculated for all demographic variables and questionnaire items. The four EIEBQ domains were analyzed individually to capture distinct emotional patterns related to eating behavior.

Internal consistency was assessed using Cronbach’s α. Associations between subscales were examined using Pearson correlation coefficients. The underlying factor structure of the EIEBQ was evaluated through Exploratory Factor Analysis (EFA). Factor scores derived from the EFA were used in subsequent analyses. Group differences across demographic variables were assessed using one-way ANOVA with post hoc comparisons where appropriate. To further explore predictors of emotional regulation in eating behavior, multiple regression models were conducted using emotional hunger awareness as the dependent variable and the four emotional mechanism indices as independent variables. Statistical significance was set at *p* < 0.05. For each emotional mechanism, item scores were averaged to create subscale indices, with higher values indicating stronger endorsement of the corresponding emotional process. These indices were subsequently used in correlation and regression analyses.

A priori power analysis was not conducted because the study was designed as an exploratory investigation of emotional mechanisms rather than a hypothesis-driven predictive model. However, post hoc evaluation indicated that the sample size (*N* = 411) provided adequate power (>0.80) to detect small effect sizes (f^2^ ≈ 0.02), consistent with the magnitude of effects observed in the final regression model. Given the exploratory nature of the study, the regression results should be interpreted as preliminary.

### 2.7. Reliability Analysis

Internal consistency of the four EIEBQ domains was evaluated using Cronbach’s α. Three subscales demonstrated acceptable to good reliability, whereas the fourth subscale (*Awareness of Emotions and Eating Behavior*) showed lower internal consistency (α < 0.50). Item–total correlations were examined to ensure that each item contributed meaningfully to its respective construct.

To further investigate the structure of this domain, a detailed psychometric inspection was conducted. Results indicated that the original five items did not form a unidimensional construct. Instead, the items clustered into two theoretically coherent components, consistent with established models of emotional awareness and emotion regulation.

Specifically:

- Items 1, 3, and 4 loaded together on a factor representing Emotional Awareness, capturing the individual’s ability to recognize emotional influences on appetite and detect changes in eating behavior across emotional states. This aligns with theoretical frameworks on interoceptive awareness and emotional monitoring.
- Items 2 and 5 loaded on a separate factor representing Emotional Eating/Emotional Overeating (Regulation), reflecting the ability to distinguish emotional from physical hunger and to regulate eating behavior through emotional understanding. This corresponds to established emotion regulation models [14] and research on cognitive control of appetite.

Although the reliability indices for the two derived components did not substantially increase, the data-driven distinction is theoretically meaningful and was retained for interpretative purposes and subsequent analyses. This refinement enhances conceptual clarity and demonstrates methodological transparency in the development of the EIEBQ. Although Cronbach’s α for the Emotional Awareness domain was lower than desirable, this is consistent with brief, heterogeneous constructs assessing emotional insight.

### 2.8. Exploratory Factor Analysis

An Exploratory Factor Analysis (EFA) was conducted to examine whether the items aligned with the four theoretically proposed domains. Sampling adequacy was evaluated using the Kaiser–Meyer–Olkin (KMO) statistic, and the suitability of the correlation matrix was assessed with Bartlett’s Test of Sphericity. Factor extraction was performed using Principal Axis Factoring, followed by oblique rotation (Promax) to allow for correlations among emotional constructs.

Factors with eigenvalues greater than 1.0 and clear interpretability were retained. Item loadings ≥ 0.40 were considered acceptable. Cross-loadings were reviewed to ensure conceptual clarity and structural coherence. The EFA confirmed the original four-factor structure, supporting the theoretical organization of the EIEBQ, while also revealing the two-component structure within the fourth domain, as described above.

### 2.9. Supplementary Material

The full English version of the Emotional Influence on Eating Behavior Questionnaire (EIEBQ) is provided in the Supplementary Materials (Table S1) to ensure transparency and replicability.

## 3. Results

The present section summarizes the sociodemographic profile of the study sample and reports the psychometric and analytical findings derived from the Emotional Influence on Eating Behavior Questionnaire (EIEBQ). Descriptive statistics are first presented to illustrate the distribution of key demographic characteristics and item-level responses. Subsequently, the dimensional structure of emotional influences on eating behavior is examined through exploratory factor analysis, followed by the construction of weighted index variables. Finally, a multiple regression model is used to investigate the extent to which these emotional mechanisms predict individuals’ ability to differentiate physical from emotional hunger.

### 3.1. Sociodemographic Characteristics of the Sample

The sociodemographic characteristics of the sample are presented in Supplementary Table S2. Most participants were female (76.6%), while males represented 23.1% of the sample; gender information was missing for 0.2% of respondents. Most participants were between 19 and 22 years old (63.3%), followed by those aged 23–28 years (19.5%) and 16–18 years (15.8%). No participants belonged to the 13–15 age group. Age information was missing for 1.5% of the sample. Regarding employment status, most respondents were full-time students (79.8%), whereas 20.2% were working students. Missing responses across demographic variables were minimal and did not materially affect the composition of the analytical sample. A small proportion of participants (1.5%) did not report their exact age; these missing values were random and showed no systematic pattern. All participants were enrolled in higher education and therefore fell within the expected developmental range of Generation Z. Consequently, the limited age non-response does not compromise the representativeness of the sample or the validity of conclusions regarding emotional eating mechanisms in this population.

### 3.2. Descriptive Statistics of Eating Behavior Items

Table 1 summarizes the descriptive statistics (Mean ± SD) for all items assessing emotional influences on eating behavior across the four dimensions of the instrument. Mean values reflect the degree of agreement with each statement on a five-point Likert scale, while standard deviations indicate inter-individual variability. Minor variations in response counts across items (*N* = 408–411) were due to sporadic missing values, and all analyses were conducted using available data per item. A detailed item-level table is provided in the Supplementary Material (Table S3).

### 3.3. Dimension-Level Interpretation of Eating Behavior

#### 3.3.1. Emotional Undereating

Participants reported moderate agreement (M = 3.10–3.27), indicating a tendency to reduce food intake in response to negative emotional states. Higher agreement was observed for eating less when feeling worried or anxious, while slightly lower agreement was reported for sadness or anger. Standard deviations (1.11–1.41) indicated substantial inter-individual variability.

#### 3.3.2. Reduced Enjoyment of Food Under Emotional Distress

Moderate agreement was also observed for this dimension (M = 3.09–3.56), suggesting that emotional states can meaningfully interfere with the sensory and hedonic aspects of eating. Difficulty enjoying food during strong emotional experiences and eating without pleasure under emotional pressure were the most strongly endorsed items. Emotional tension reducing the ability to taste food showed the greatest variability (SD = 1.92). This item showed the highest variability, indicating substantial individual differences in sensory responses to emotional tension.

#### 3.3.3. Emotional Eating/Emotional Overeating

This dimension showed low to moderate agreement (M = 2.61–2.98), indicating that participants did not strongly endorse using food as a primary emotional regulation strategy. The highest agreement was observed for using food to calm down when stressed or sad, while the lowest was reported for coping with difficult emotions.

#### 3.3.4. Emotional Awareness and Control in Eating

Participants demonstrated consistently high levels of emotional awareness (M = 3.42–4.06), reflecting strong recognition of how emotions influence eating patterns. The highest agreement was observed for noticing appetite changes depending on emotional state.

### 3.4. Factor Structure of Eating Behavior

An exploratory factor analysis (EFA) was conducted to examine the latent structure of eating behavior. Sampling adequacy was excellent (KMO = 0.90), and Bartlett’s test of sphericity was significant (χ^2^ (153) = 3450.12, *p* < 0.001), confirming suitability for factor analysis.

The analysis yielded a four-factor solution, explaining 63.23% of the total variance. The factors were interpreted as:

1. Emotional Undereating
2. Reduced Enjoyment of Food under Emotional Distress
3. Emotional Eating/Emotional Overeating
4. Emotional Awareness and Control in Eating

The four-factor solution demonstrated strong conceptual coherence and aligned with the theoretically proposed structure of the EIEBQ.

A summary of primary factor loadings and cross-loadings above 0.30 is presented in Table 2, while the full factor loading matrix is provided in Supplementary Table S4. Cross-loadings were minimal, with only one item (Q2.4) showing a secondary loading above 0.30 (–0.465 on EO), which did not exceed its primary loading on Reduced Enjoyment (0.421).

For clarity, the four extracted factors are abbreviated as follows: EO = Emotional Overeating, RE = Reduced Enjoyment of Food under Emotional Distress, EA = Emotional Awareness, and EU = Emotional Undereating. These abbreviations are used consistently in both the summary table presented here and the full factor loading matrix in Supplementary Table S4.

Bivariate correlations among the four emotional mechanisms were generally small to moderate (r = 0.10–0.42). Emotional Awareness showed the lowest correlations with the other indices (r = 0.10–0.21), suggesting a weaker association with the behavioral components of emotional eating. However, given the low internal consistency of the Emotional Awareness factor (α = 0.487), these attenuated correlations may partly reflect measurement unreliability rather than true discriminant validity. Therefore, the observed pattern should be interpreted with caution.

An exploratory factor analysis (EFA) was conducted to examine the latent structure of eating behavior. Sampling adequacy was excellent (KMO = 0.90), and Bartlett’s test of sphericity was significant (χ^2^(153) = 3450.12, *p* < 0.001). The analysis yielded a four-factor solution explaining 63.23% of the total variance, corresponding to Emotional Undereating, Reduced Enjoyment, Emotional Overeating, and Emotional Awareness. One item (EA5) was removed due to low communality and conceptually inconsistent loading. Internal consistency was strong for three factors (α = 0.793–0.877), while the Emotional Awareness factor showed lower reliability (α = 0.487). A detailed item-level factor loading matrix is provided in the Supplementary Material (Table S4).

### 3.5. Descriptive Statistics and Correlations Among Index Variables

Descriptive statistics and bivariate correlations for the four weighted index variables derived from the EFA—Emotional Undereating, Reduced Enjoyment, Emotional Overeating, and Emotional Awareness—are presented in Table 3. All indices demonstrated adequate variability, with mean values ranging from 0.57 to 0.78 and standard deviations between 0.13 and 0.19, indicating sufficient dispersion for subsequent predictive analyses.

As expected, the indices were significantly intercorrelated, reflecting theoretically coherent but distinct emotional mechanisms. Emotional Undereating showed moderate positive correlations with both Reduced Enjoyment (*r* = 0.50, *p* < 0.01) and Emotional Overeating (*r* = 0.43, *p* < 0.01), suggesting shared emotional reactivity patterns in eating behavior. Reduced Enjoyment was also moderately associated with Emotional Overeating (*r* = 0.47, *p* < 0.01). Emotional Awareness demonstrated small but significant correlations with the other indices (*r* = 0.10–0.21, *p* < 0.05), indicating that awareness of emotional influences on eating is related to, yet partially independent from, emotional reactivity and regulation tendencies.

These findings confirm that the four indices capture interconnected but non-overlapping constructs, supporting their suitability for inclusion in the subsequent regression models predicting emotional hunger awareness.

### 3.6. Regression Model Predicting Emotional Hunger Awareness

A multiple linear regression analysis was conducted to examine the extent to which the four emotional-mechanism indices predicted participants’ ability to distinguish physical from emotional hunger. The overall model was statistically significant, *F*(4, 405) = 4.427, *p* = 0.002, explaining 4.2% of the variance in emotional hunger awareness (Table 4). Although the effect size was small, the model provides meaningful insight into the emotional processes underlying hunger differentiation. Although the explained variance was modest, the model revealed meaningful behavioral pathways underlying hunger differentiation.

Among the predictors, Emotional Undereating emerged as a significant positive predictor (*β* = 0.155, *p* = 0.017), indicating that individuals who tend to reduce food intake under negative emotional states are more likely to accurately identify emotional hunger. Emotional Overeating also significantly predicted hunger awareness (*β* = 0.135, *p* = 0.027), suggesting that heightened emotional reactivity in eating—whether expressed as eating more or less—enhances recognition of emotional cues.

In contrast, Reduced Enjoyment (*β* = 0.054, *p* = 0.342) and Emotional Awareness Index (*β* = 0.079, *p* = 0.132) were not significant predictors in the final model. These findings indicate that behavioral manifestations of emotional reactivity (overeating and undereating) play a more central role in hunger differentiation than cognitive awareness alone.

Although statistically significant, the overall explanatory power of the regression model was modest (R^2^ = 0.042), indicating that the four emotional mechanisms accounted for only a small proportion of variance in emotional hunger awareness. This limited effect suggests that additional psychological, contextual, or behavioral factors likely contribute to this outcome.

It should be noted that the Emotional Awareness factor demonstrated low internal consistency (α = 0.487), which likely attenuated its association with emotional hunger awareness. Therefore, the non-significant regression coefficient for this predictor should be interpreted cautiously, as the true relationship may be underestimated due to measurement unreliability.

Demographic variables (sex, age, employment status) were examined in preliminary analyses using independent-samples *t*-tests and one-way ANOVA. None of the demographic variables showed statistically significant associations with emotional hunger awareness (all *p* > 0.10), nor did they improve model fit when entered as covariates (ΔR^2^ < 0.01). Based on these results, demographic variables were excluded from the final regression model to maintain parsimony. Because the sample was highly unbalanced by sex, moderation by sex was also tested; however, interaction terms were nonsignificant (*p* > 0.10), indicating no moderating effect.

**Figure 1:**
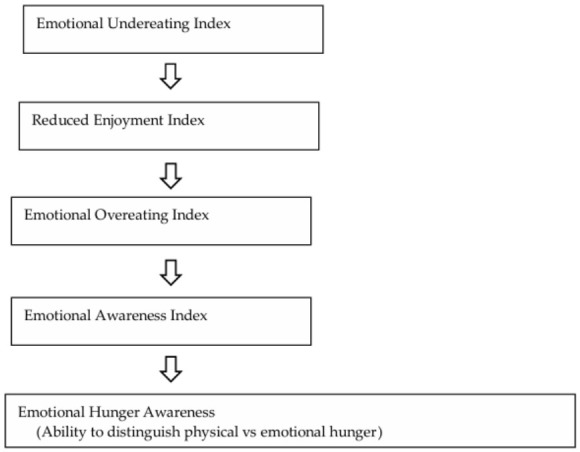
Conceptual framework of the predictive model of emotional hunger awareness.

Figure 1 illustrates the conceptual framework of the predictive model, depicting how the four emotional mechanisms—emotional undereating, reduced enjoyment, emotional overeating, and emotional awareness—are hypothesized to influence emotional hunger awareness. Each mechanism is represented as a weighted index derived from the EIEBQ, reflecting the proposed directionality of emotional processes that shape individuals’ ability to differentiate physical from emotional hunger.

**Figure 2:**
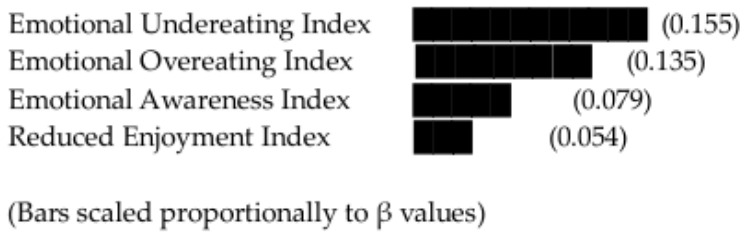
Standardized beta coefficients from the multiple regression model predicting emotional hunger awareness. Bars represent the relative strength of each emotional mechanism, with higher values indicating stronger predictive influence. Note: Error bars represent 95% confidence intervals for each regression coefficient.

Figure 2 presents the standardized beta coefficients from the multiple regression model, highlighting the relative contribution of each emotional mechanism to emotional hunger awareness. Emotional Undereating (β = 0.155) and Emotional Overeating (β = 0.135) emerged as modest but statistically significant predictors, whereas Emotional Awareness (β = 0.079) and Reduced Enjoyment (β = 0.054) showed weaker, non-significant effects. Visualization underscores that behavioral emotional responses exert a stronger influence on hunger differentiation than cognitive awareness.

## 4. Discussion

### 4.1. Summary of Key Findings

This study examined emotional influences on eating behavior among young adults and identified four distinct mechanisms—emotional undereating, reduced enjoyment, emotional overeating, and emotional awareness. Participants demonstrated moderate emotional undereating of food intake and reduced enjoyment, low to moderate use of food for emotion regulation, and consistently high emotional awareness. The factor structure showed strong psychometric coherence for three of the four dimensions, supporting the validity of the proposed model.

The exploratory factor analysis confirmed a clear four-factor structure with satisfactory communalities and high internal consistency for most factors. One item from the Emotional Awareness subscale (EA5: *“Understanding my emotions helps me regulate my eating behavior”*) was removed due to inadequate psychometric performance. Although conceptually relevant, the item loaded weakly and inconsistently on the Emotional Undereating factor and demonstrated very low communality, indicating that it did not align with the latent construction captured by the remaining Emotional Awareness items. Similar findings have been reported in validations of emotion-related eating measures, where items reflecting general emotional insight rather than eating-specific awareness tend to perform poorly. Removing EA5 improved the structural clarity and internal consistency of the Emotional Awareness factor, resulting in a more coherent and interpretable model.

Importantly, emotional undereating and emotional overeating emerged as the strongest predictors of emotional hunger awareness, whereas reduced enjoyment and emotional awareness did not significantly contribute to the predictive model. Overall, the refined four-factor structure provides a useful preliminary framework for understanding how emotional processes shape eating behavior, highlighting the multidimensional nature of emotional influences on food-related decisions.

Despite the statistical significance of emotional undereating and emotional overeating as predictors, the overall explanatory power of the regression model was low (R^2^ = 4.2%). This indicates that the four emotional mechanisms accounted for only a small proportion of variance in emotional hunger awareness. Emotional hunger is likely shaped by additional psychological, contextual, and interoceptive factors—such as stress reactivity, personality traits, coping styles, and physiological sensitivity—which have been consistently linked to emotional eating and hunger misperception in prior research [12,29,30,31,32,33]. These mechanisms were not assessed in the present study, and therefore the predictive findings should be interpreted with caution.

The low internal consistency of the Emotional Awareness subscale (α = 0.487) warrants further consideration. Several factors may account for this reduced reliability, including the small number of items, the removal of EA5 due to low communality, and the known variability in emotional introspection among young adults. Emotional awareness items often show weaker psychometric performance in youth samples, particularly when they require abstract reflection rather than concrete behavioral descriptions. Cultural and linguistic nuances in the interpretation of emotional terms may also have contributed to inconsistent responses. These limitations suggest that the current item pool may not fully capture the construct of eating-specific emotional awareness.

Although statistically significant, these associations were modest in magnitude. Therefore, the findings should be interpreted as indicative rather than conclusive, reflecting early patterns that require further investigation. For clarity and transparency, the graphical representation of these regression coefficients (Figure 2) has been updated to include 95% confidence intervals, providing a more accurate depiction of estimate precision and uncertainty.

Beyond their theoretical relevance, these findings have direct implications for school nursing and university health services. The identification of distinct emotional response patterns—particularly emotional undereating and emotional overeating—provides a practical framework for early detection of students who may struggle to differentiate emotional from physical hunger. School and university nurses are often the first point of contact for young adults experiencing stress-related changes in appetite, and the mechanisms identified in this study can support brief screening, targeted conversations, and referral pathways. Interventions that focus on helping students recognize physiological hunger cues, monitor emotional triggers, and distinguish emotional from physical appetite may enhance self-regulation and reduce maladaptive eating responses. Incorporating psychoeducation, emotion-recognition training, and structured monitoring of physical hunger patterns into campus health programs could strengthen preventive care and promote healthier eating behaviors among young adults.

### 4.2. Emotional–Eating Patterns in Generation Z

Nearly all participants belonged to Generation Z, a cohort widely characterized as highly digitally immersed and routinely exposed to rapid information flows and online food-related content [34,35]. Prior research indicates that such digital environments can shape attentional patterns, emotional processing, and food-related decision-making in young people. These contextual characteristics are provided solely to aid interpretation and were not measured in the present study.

Across all dimensions, the results reveal a distinct emotional–eating profile:

- **Emotional Undereating:** This pattern is consistent with evidence showing that younger generations report heightened psychological pressure and emotional volatility during the post-pandemic period, conditions that can contribute to somatic manifestations such as reduced appetite [36,37].
- **Reduced Enjoyment of Food under Emotional Distress:** Strong emotional activation disrupted hedonic engagement with food. Difficulty savoring food and eating without pleasure were common, consistent with evidence that emotional overload can blunt sensory responsiveness during eating. Similar patterns of reduced hedonic engagement under negative effect have been documented among adolescents and young adults, who often show heightened emotional reactivity [29,38,39].
- **Emotional Eating/Emotional Overeating:** This pattern is consistent with evidence indicating that Generation Z frequently relies on cognitive or digital coping strategies—such as online distraction and peer communication—rather than traditional comfort-eating responses [40,41].
- **Emotional Awareness and Control in Eating:** This pattern reflects a broader developmental phenomenon, as extensive evidence shows that emotional insight does not necessarily translate into effective behavioral regulation during adolescence and emerging adulthood [13,42,43].

Taken together, these patterns reflect a multifaceted emotional–eating profile shaped by psychosocial stressors and the developmental demands of emerging adulthood, within a broader digitalized social context [29,44].

These patterns should be interpreted cautiously, as they represent tendencies rather than strong effects, consistent with the modest associations observed in the predictive analyses.

Moreover, because the sample was predominantly female, the emotional–eating patterns described here should not be interpreted as representative of Generation Z as a whole. These patterns may differ substantially in male or gender-diverse subgroups, and the present study was not designed to test such differences.

### 4.3. Interpretation Considering Existing Literature

The findings align with studies showing that the COVID-19 period intensified emotional reactivity in eating behaviors. Both emotional overeating and undereating have been documented as stress-related responses during periods of uncertainty [45,46]. Comparable patterns of appetite suppression under negative effect have been documented in young-adult samples, reinforcing the present study’s finding that emotional undereating represents a meaningful behavioral response within Generation Z [29,47]. These interpretations should not be taken to imply that generational traits were measured or tested as explanatory variables in the present study.

The disruption of hedonic engagement observed in this sample is consistent with research demonstrating that emotional distress reduces sensory responsiveness and food enjoyment [45]. These findings reinforce the notion that strong emotional activation can blunt hedonic processing, leading to diminished pleasure during eating episodes.

The limited reliance on food as an emotion-regulation strategy aligns with recent evidence indicating that younger cohorts increasingly adopt cognitive or digital coping mechanisms rather than traditional comfort eating [48,49]. This shift may explain the relatively low levels of emotional overeating observed in the present study.

The non-significant role of reduced enjoyment and emotional awareness is theoretically meaningful. Although emotional awareness is often conceptualized as protective, its limited predictive value suggests that awareness alone is insufficient for accurate hunger differentiation [50,51]. One possible interpretation is that behavioral learning—accumulated through repeated experiences of eating under different emotional states—may gradually enhance or impair hunger differentiation, a process consistent with evidence on emotional-eating patterns and interoceptive sensitivity [33,47]. However, this explanation is speculative and was not directly tested in the present study. An equally plausible alternative is that the non-significant effect of emotional awareness reflects the limited internal consistency of this subscale, which may have reduced its ability to detect a true association. Therefore, no firm conclusions can be drawn regarding the relative influence of behavioral learning versus introspective clarity, and future research is needed to examine these mechanisms more rigorously [17,19].

Despite its low reliability, the Emotional Awareness factor was retained in the predictive model due to its theoretical relevance. Emotional awareness is a core component in established frameworks of emotional eating, and excluding it would have resulted in an incomplete representation of the emotional mechanisms under investigation. Nevertheless, the low reliability likely attenuated its predictive effect, and the corresponding estimates should be interpreted with caution.

Overall, the present findings contribute to existing literature by suggesting that behavioral emotional responses—rather than cognitive insight—are the strongest predictors of emotional hunger awareness in young adults.

### 4.4. Theoretical Implications

This study offers several theoretical contributions:

- Behavioral emotional reactivity (overeating and undereating) appears more closely associated with hunger differentiation than cognitive or sensory mechanisms.
- The four-factor structure provides a refined conceptualization of emotional eating, distinguishing between hedonic disengagement and behavioral inhibition.
- These findings point to a potential mismatch between emotional literacy and behavioral regulation, a developmental pattern frequently observed in adolescence and emerging adulthood, including Generation Z [13,42].

The conceptual model developed in this study integrates these components, illustrating how emotional sensitivity, hedonic disruption, selective coping tendencies, and emotional awareness jointly contribute to shaping eating behavior, in line with established evidence on emotion-regulation and coping processes [12,52].

However, given the modest effect sizes, this conceptual model should be viewed as preliminary and subject to further empirical refinement.

### 4.5. Practical Implications

Evidence indicates that behavioral components—such as self-monitoring, stimulus control, and behavioral activation—play a central role in reducing dysregulated eating and may be particularly beneficial for individuals prone to emotion-driven eating [53,54]. Comparative findings further show that behavioral and acceptance-based approaches often outperform purely cognitive strategies in enhancing eating-related self-regulation [55,56,57]. These implications are provided solely as contextual considerations and were not directly tested in the present study.

Behavior-focused interventions may be more effective than purely cognitive approaches. Helping individuals recognize behavioral patterns (e.g., eating less when anxious) may enhance hunger differentiation.

- University-based services could integrate screening tools that capture both overeating and undereating tendencies.
- Post-pandemic recovery programs may benefit from addressing emotional reactivity in eating, given statistically significant but modest association with hunger awareness.
- Digital-based interventions may be well suited for Generation Z, as younger cohorts frequently rely on online communication, mobile platforms, and digital environments for emotional coping and self-regulation [40]. Evidence also shows that digital health interventions are highly acceptable and effective among adolescents and young adults [58]. These implications are provided solely as contextual considerations and were not directly assessed in the present study [40,58,59].

### 4.6. Strengths and Limitations

Strengths of the study include the large sample size, the use of a multidimensional instrument grounded in established emotional-eating frameworks, and the strong factor-analytic support for the proposed model. The predictive analysis provides novel insights into the behavioral mechanisms underlying emotional hunger awareness, highlighting the potential relevance of emotional reactivity in distinguishing emotional from physical hunger.

Although demographic variables were examined in preliminary analyses, none showed significant associations with emotional hunger awareness (all *p* > 0.10), nor did they improve model fit (ΔR^2^ < 0.01). Interaction terms testing sex as a moderator were also nonsignificant. Given these results, demographic variables were excluded from the final regression model to maintain parsimony. Nevertheless, the highly unbalanced gender distribution limits the strength of this conclusion, and future studies should examine demographic moderators using more balanced samples. These findings should be interpreted as preliminary and exploratory.

A major limitation of the present study concerns the gender composition of the sample, which was predominantly female (76.6%). This imbalance substantially restricts the generalizability of the findings to the broader Generation Z population. Recent evidence highlights pronounced gender differences in emotional eating, emotion-regulation strategies, and the pathways linking affect dysregulation to disordered eating, with women consistently showing higher emotional and external eating scores and distinct regulatory profiles [60,61,62]. Given these well-established sex-specific patterns, the absence of gender-stratified analyses or formal moderation testing in the present study means that the predictive relationships identified—particularly those involving emotional reactivity—cannot be assumed to operate equivalently across genders. Future research should explicitly examine gender as a moderator and report gender-specific descriptive and predictive results to ensure that conclusions reflect the heterogeneity of emotional eating within Generation Z.

In addition, demographic characteristics (e.g., gender, age, employment status) were reported solely to describe the University of Ioannina student sample and were not examined as correlates or predictors of emotional-eating indices. As a result, the present study does not assess whether emotional-eating patterns differ across demographic subgroups. This analytic choice further limits the generalizability of the findings, and future research should incorporate demographic comparisons and moderator analyses to determine whether the emotional mechanisms identified operate similarly across different segments of Generation Z.

A linguistic consideration also warrants mention. The questionnaire did not provide definitions for emotional terms (e.g., irritation, worry, anxiety, sadness, anger), allowing participants to interpret them according to their own emotional perception. In Greek, certain emotional terms—such as ‘άγχος’ (anxiety) and ‘ανησυχία’ (worry)—carry distinct affective and psycholinguistic profiles, with the former typically associated with higher arousal and somatic intensity and the latter with milder, cognitively oriented concern [63]. Although such nuances may influence how respondents interpret emotional triggers, the factor-analytic structure of the EIEBQ indicated that participants meaningfully differentiated among the emotional constructs. Nonetheless, future studies should consider cognitive interviewing or pilot testing to further examine potential linguistic effects on item interpretation.

Limitations include the cross-sectional design, reliance on self-reported measures, and the lower reliability observed in the Emotional Awareness factor. An important implication of this low reliability is that the predictive association between Emotional Awareness and emotional hunger awareness is likely underestimated. Measurement error attenuates regression coefficients, increasing the probability of Type II error and reducing the ability to detect true effects. Therefore, the non-significant finding for Emotional Awareness should not be interpreted as evidence of a negligible or absent relationship, but rather as a result that must be viewed with caution given the psychometric limitations of the subscale. This factor requires refinement, additional item development, and validation in independent samples before firm conclusions can be drawn regarding its role in emotional eating processes. The sample consisted primarily of university students, which may limit generalizability to broader youth populations. Future research should employ longitudinal or experimental designs to examine how emotional-eating patterns evolve over time and whether modifying behavioral responses improves hunger differentiation.

Future studies should expand and refine the Emotional Awareness subscale by developing new items that more directly assess eating-specific emotional insight, integrating both interoceptive and cognitive components. Cognitive interviewing and pilot testing could help identify sources of misinterpretation, while larger and more diverse samples may improve item discrimination. Revising or replacing items with low factor loadings and increasing the number of items may enhance internal consistency and structural coherence.

Moreover, the cross-sectional design of the study precludes any causal interpretation of the observed associations. Although the regression model identifies statistical predictors of emotional hunger awareness, these relationships cannot be taken to imply temporal or mechanistic causality. The direction of effects remains unknown, and it is equally plausible that emotional hunger awareness influences behavioral responses or that both are shaped by unmeasured third variables. Longitudinal and experimental research is required to determine whether behavioral emotional responses exert a causal influence on hunger differentiation over time.

The study did not include priori power analysis. Although the large sample size (*N* = 411) provides adequate power to detect small effects, the low explained variance in the regression model (R^2^ = 4.2%) indicates that the predictive findings should be interpreted with caution. Future studies should incorporate formal power calculations based on expected effect sizes to ensure adequate sensitivity for detecting associations of this magnitude.

Beyond statistical significance, the explanatory strength of the model is substantively weak, as 4.2% explained variance reflects only a minimal contribution of the assessed emotional mechanisms to emotional hunger awareness. This limitation underscores the need for future models to incorporate additional psychological, contextual, and interoceptive variables to more fully account for the determinants of emotional hunger.

In addition to these psychological and interoceptive mechanisms, several other categories of determinants may substantially influence emotional hunger awareness but were not assessed in the present study. Biological factors such as cortisol reactivity, autonomic arousal, hormonal fluctuations, and interoceptive accuracy have been shown to shape appetite regulation and emotional eating tendencies. Socioeconomic conditions—including financial strain, food insecurity, academic pressure, and environmental stress exposure—may also contribute to heightened emotional vulnerability around eating. Furthermore, mental health-related variables such as anxiety, depressive symptoms, emotion dysregulation, and trauma history are consistently associated with dysregulated eating patterns and may account for additional variance in emotional hunger. The omission of these domains likely contributed to the low explanatory power of the model, and future research should incorporate these biological, socioeconomic, and mental-health factors to develop a more comprehensive understanding of emotional hunger.

A further limitation concerns the gender composition of the sample. Although sex-stratified regression analyses were considered, the very small male subsample (*n* = 95) precluded the estimation of stable or interpretable models. Running separate regressions under such conditions would yield unreliable coefficients, inflated standard errors, and spurious patterns. For this reason, no stratified analysis was conducted. Instead, the findings were contextualized using existing literature on male emotional eating, which consistently reports lower emotional reactivity, reduced emotional overeating, and distinct coping tendencies among men compared with women. These discrepancies underscore the need for future studies with more balanced gender distributions to examine sex-specific mechanisms more rigorously. Descriptive sensitivity analyses stratified by sex are provided in Supplementary Tables S5 and S6, presenting item- and subscale-level comparisons to illustrate preliminary gender-related patterns.

Another methodological limitation concerns the handling of missing data. Although missingness was minimal and appeared random, the use of pairwise deletion may introduce small estimation biases compared to more advanced methods such as multiple imputation or full information maximum likelihood. Future studies should consider these approaches, particularly when working with larger or more heterogeneous datasets.

An additional methodological limitation concerns the use of the same dataset for both the initial exploratory psychometric evaluation of the EIEBQ and the substantive analyses examining emotional mechanisms of eating behavior. Although this approach is acceptable for preliminary, hypothesis-generating research, it does not constitute a full validation of the instrument. Future studies should employ independent samples, split-sample designs, or cross-validation procedures to confirm the stability of the factor structure and to ensure that behavioral associations are not sample-specific.

In addition to these considerations, an important methodological aspect concerns the instrument itself.

A notable strength of this study is the introduction and initial psychometric evaluation of the Emotional Influence on Eating Behavior Questionnaire (EIEBQ), a theoretically grounded and behavior-focused instrument derived from our previous research program on post-pandemic eating behavior. Although the EIEBQ is newly developed and this study represents its first systematic validation, the instrument is firmly anchored in established models of emotional eating and informed by widely used measures such as the DEBQ, EES, TFEQ, and AEBQ. The use of a large sample and a transparent, data-driven factor-analytic approach further support its structural coherence. Nevertheless, as with any newly developed tool, additional validation in diverse populations and cultural contexts is warranted. Future studies should examine test–retest reliability, convergent validity with established emotional eating scales, and potential measurement invariance across demographic groups. Furthermore, the low internal consistency of the Emotional Awareness factor (α = 0.487) may have attenuated its predictive association with emotional hunger awareness, leading to an underestimation of its true effect. This attenuation bias should be considered when interpreting the non-significant regression coefficient, and future studies should refine this subscale to improve reliability. In addition, the removal of item EA5 due to low communality highlights the need for conceptual and linguistic refinement of the Emotional Awareness subscale. Future research should reformulate or expand the item pool to ensure clearer alignment with the underlying construction and to enhance both reliability and structural coherence. Despite these considerations, the present findings provide initial preliminary evidence for the EIEBQ as a conceptually robust and practically useful instrument for assessing emotional mechanisms of eating behavior in young adults. The use of a five-point Likert scale is consistent with established emotional-eating measures and provides adequate variability for robust psychometric evaluation.

A further limitation concerns the content-validation process. Because no expert panel, pilot testing, or cognitive interviewing was conducted, the content validity of the EIEBQ should be considered preliminary. Future research should employ formal content-validation procedures and evaluate the psychometric properties of both the parent scale and the derived instrument in independent samples.

It is also important to note that the present study provides the first empirical evaluation of the EIEBQ within a Greek young-adult population, offering initial evidence for its cultural applicability and supporting its use in future research and public-health settings in Greece.

The highly unbalanced gender distribution (76.6% female) also prevented reliable sex-stratified regression analyses. The small male subsample would yield unstable estimates and inflated standard errors, limiting the interpretability of any subgroup model. As a result, gender-specific trends could not be meaningfully explored, and the present findings should not be generalized to male or gender-diverse populations. Future research should recruit more balanced samples to enable robust examination of sex-related differences in emotional eating mechanisms.

### 4.7. Overall Contribution

Overall, this study advances understanding of emotional eating by demonstrating that behavioral emotional responses—rather than cognitive awareness—may be more closely associated of emotional hunger awareness. The integration of psychometric evidence, behavioral patterns, and generational context provides a preliminary integrative perspective for interpreting emotional influences on eating in young adults. These findings highlight potential targets for intervention among young adult women, while underscoring the need for gender-inclusive research to determine whether similar patterns emerge across the broader Generation Z population.

It is important to note that the generational characteristics discussed in this section (e.g., digital immersion, online coping tendencies, rapid information exposure) were not directly measured in the present study. These contextual descriptions are intended to situate the findings within broader sociocultural trends rather than to serve as explanatory variables. Therefore, any generational interpretation should be viewed as speculative and not as empirically tested mechanisms within this dataset.

## 5. Conclusions

This study provides an initial, exploratory examination of emotional influences on eating behavior among young adults using a newly developed instrument. The identification of a four-factor structure—emotional undereating, reduced enjoyment, emotional overeating, and emotional awareness—offers a preliminary framework for conceptualizing the multidimensional ways in which emotional states relate to eating patterns. While the factor structure demonstrated acceptable coherence for most dimensions, this represents the first validation of the instrument, and therefore the psychometric findings should be interpreted as provisional rather than definitive.

A key finding of the study is that behavioral emotional responses—specifically emotional undereating and emotional overeating—emerged as the strongest predictors among the variables examined. However, these predictors collectively accounted for only 4.2% of the variance in emotional hunger awareness, indicating that their practical influence is modest. As such, the results suggest a potential, rather than a dominant, role for behavioral reactivity in hunger differentiation, and they should not be interpreted as evidence of strong or exclusive mechanisms.

The study also highlights the broader psychosocial context in which emotional–eating tendencies develop, although these contextual factors were not directly measured and therefore cannot be considered explanatory variables. Understanding how young adults navigate emotional cues, stressors, and food-related decisions remains an important direction for future research.

Future studies should employ longitudinal and experimental designs to clarify causal pathways, evaluate whether modifying emotional eating behaviors improves hunger differentiation, and examine whether these patterns hold across genders and cultural contexts. Additional psychometric work is also needed to further validate the instrument, refine the Emotional Awareness subscale, and assess measurement invariance.

Overall, this study offers a preliminary foundation for future research on emotional eating in young adults. By integrating early psychometric evidence with behavioral findings, it provides an initial step toward developing more comprehensive models and targeted interventions that support healthier eating patterns.

## Figures and Tables

**Table 1:** Mean values and standard deviations for the four EIEBQ subscales. Higher scores indicate stronger endorsement of each emotional eating mechanism.

Subscale	Item Codes	Mean Value *	Standard Deviation
Emotional Undereating	Q1.1–Q1.5	3.17	1.26
Reduced Enjoyment of Food under Emotional Distress	Q2.1–Q2.5	3.38	1.24
Emotional Eating/Emotional Overeating	Q3.1–Q3.5	2.86	1.18
Emotional Awareness and Control in Eating	Q4.1–Q4.5	3.76	0.93

* Note: Values range from one to five.

**Table 2:** Summary of primary factor loadings and cross-loadings (>0.30) from the Exploratory Factor Analysis. The complete factor loading matrix is available in Supplementary Table S4.

Item Code	Primary Loading	Factor	Cross-Loadings (>0.30)
Q1.1	0.851	EU	–
Q1.2	0.620	EO (negative loading)	–
Q1.3	0.645	EO (negative loading)	–
Q1.4	0.471	EO (negative loading)	–
Q1.5	0.847	EU	–
Q2.1	0.823	RE	–
Q2.2	0.831	RE	–
Q2.3	0.784	RE	–
Q2.4	0.421	RE	–0.465 (EO)
Q2.5	0.721	RE	–
Q3.1	0.788	EO	–
Q3.2	0.798	EO	–
Q3.3	0.819	EO	–
Q3.4	0.761	EO	–
Q3.5	0.754	EO	–
Q4.1	0.566	EA	–
Q4.2	0.733	EA	–
Q4.3	0.788	EA	–
Q4.4	0.703	EU	–
Q4.5	0.440	EU	–

**Table 3:** Descriptive Statistics and Correlations Among Index Variables.

Variable	Mean	SD	1	2	3	4
1. Emotional Undereating Index	0.63	0.18	-	0.502 **	0.430 **	0.118 **
2. Reduced Enjoyment of Food under Emotional Distress Index	0.67	0.17	0.502 **	-	0.469 **	0.099 *
3. Emotional Eating/Emotional Overeating Index	0.57	0.19	0.430 **	0.469 **	-	0.210 **
4. Emotional Awareness and Control in Eating Index	0.78	0.13	0.118 **	0.099 *	0.210 **	-

**Note.** *N* = 410. Pearson correlations (two-tailed). * *p* < 0.05, ** *p* < 0.01.

**Table 4:** Multiple Regression Model Predicting Emotional Hunger Awareness (*N* = 410).

Predictor	B	SE	β	t	*p*
Emotional Undereating Index	0.790	0.330	0.155	2.395	0.017
Reduced Enjoyment Index	0.302	0.317	0.054	0.952	0.342
Emotional Overeating Index	0.651	0.294	0.135	2.214	0.027
Emotional Awareness Index	0.551	0.365	0.079	1.508	0.132
Constant	1.909	0.369	-	5.176	

**Model fit:** R = 0.205, R^2^ = 0.042, Adjusted R^2^ = 0.032, F(4, 405) = 4.427, *p* = 0.002. Statistical Notes: B (Unstandardized Coefficient), SE (Standard Error), β (Standardized Beta Coefficient), t (*t*-value), *p* (*p*-value).

## Data Availability

The data presented in this study is available on request from the corresponding author due to legal reasons.

## Supplementary Materials

The following supporting information can be downloaded at: https://www.mdpi.com/article/10.3390/nu18101500/s1, Table S1: Questionnaire on Emotional Influence on Consumers’ Eating Behavior; Table S2: Sociodemographic characteristics of the study sample (*N* = 411); Table S3: Mean values and standard deviations for all items assessing emotional influences on eating behavior; Table S4: Exploratory factor analysis results for the four-factor model of post-COVID eating behavior (*N* = 411); Table S5. Item-level descriptive statistics (Mean ± SD) for all 20 emotional-eating items, stratified by gender; Table S6. Mean ± SD scores for the four emotional-eating subscales (Emotional Undereating, Reduced Enjoyment, Emotional Eating, Emotional Awareness) by gender.
