# Supplementary material for: Emotional Influences on Eating Behavior and Hunger Awareness Among Generation Z University Students in Greece
Source: Nutrients. 2026 May 8;18(10):1500. doi: 10.3390/nu18101500 (PMC13209663; doi:10.3390/nu18101500)
Supplement: Supplementary file 1 [file nutrients-18-01500-s001.zip › nutrients-4295735-supplementary.pdf]

nutrients

Article

Emotional Influences on Eating Behavior and Hunger Awareness Among Generation Z University Students in Greece

Maria P. Koliou [1], Chrysoula Karaiskou [2], Charalampos Eleftheriadis [2], Achilleas Kontogeorgos [3] and Dimitris Skalkos [1,*]

[1] Laboratory of Food Chemistry, Department of Chemistry, University of Ioannina, 45110 Ioannina, Greece; m.koliou@uoi.gr
[2] Sidroco Holdings Ltd., Nicosia 1082, Cyprus; ckaraiskou@sidroco.com (C.K.); celeftheriadis@sidroco.com (C.E.)
[3] Department of Agriculture, International University of Greece, 57001 Thessaloniki, Greece; akontoge@ihu.gr
* Correspondence: dskalkos@uoi.gr; Tel.: +30-2651008345

Supplementary Material

The Supplementary Materials include the full questionnaire and detailed psychometric tables supporting the analyses presented in the manuscript.

Table S1. Questionnaire on Emotional Influence on Consumers' Eating Behavior.

I. Demographic Information

1. Gender
Male
Female

2. Age
13-15
16-18
19-22
23-28

3. Employment / Study Status
Working student
Full-time student

4. Region of permanent residence in Greece
Northern Greece (Macedonia – Thrace)
Western Greece (Epirus – Aetolia-Acarnania)
Central Greece (including Athens)
Peloponnese
Islands (Aegean and Ionian)

Academic Editor: Joseph Sharkey

Received: 17 April 2026
Revised: 30 April 2026
Accepted: 6 May 2026
Published: 8 May 2026

Copyright: © 2026 by the authors. Licensee MDPI, Basel, Switzerland. This article is an open access article distributed under the terms and conditions of the Creative Commons Attribution (CC BY) license.

Emotional Influence on Consumers' Eating Behavior

Please indicate your level of agreement with each statement using a 5-point Likert scale ranging from **1 = Strongly Disagree** to **5 = Strongly Agree**. A score of **1** represents **strong disagreement** with the statement, while a score of **5** represents **complete agreement**. A score of **3** indicates a **neutral response**, due to uncertainty or ambivalence.

(Please mark your answer with an "X".)

1. Emotional Undereating

Compared to my usual behavior	Strongly Disagree	Disagree	Neutral	Agree	Strongly Angree
1. I eat less when I am irritated.	☐ 1	☐ 2	☐ 3	☐ 4	☐ 5
2. I eat less when I feel worried.	☐ 1	☐ 2	☐ 3	☐ 4	☐ 5
3. I eat less when I feel anxious.	☐ 1	☐ 2	☐ 3	☐ 4	☐ 5
4. I eat less when I feel sad.	☐ 1	☐ 2	☐ 3	☐ 4	☐ 5
5. I eat less when I am angry.	☐ 1	☐ 2	☐ 3	☐ 4	☐ 5

2. Reduced Enjoyment of Food under Emotional Distress

Compared to my usual behavior	Strongly Disagree	Disagree	Neutral	Agree	Strongly Agree
1. When I experience strong emotions, I have difficulty enjoying food.	☐ 1	☐ 2	☐ 3	☐ 4	☐ 5
2. Emotional tension reduces my enjoyment of food.	☐ 1	☐ 2	☐ 3	☐ 4	☐ 5
3. When I am emotionally upset, food tastes less pleasant to me.	☐ 1	☐ 2	☐ 3	☐ 4	☐ 5
4. Negative emotions make me lose interest in eating.	☐ 1	☐ 2	☐ 3	☐ 4	☐ 5
5. When I feel emotionally pressured, I eat without pleasure.	☐ 1	☐ 2	☐ 3	☐ 4	☐ 5

3. Emotional Eating/ Emotional Overeating

Compared to my usual behavior	Strongly Disagree	Disagree	Neutral	Agree	Strongly Agree
1. I feel a desire to eat when I am stressed.	☐ 1	☐ 2	☐ 3	☐ 4	☐ 5
2. When I feel tense, I have a strong urge to eat.	☐ 1	☐ 2	☐ 3	☐ 4	☐ 5
3. I use food as a way to cope with difficult emotions.	☐ 1	☐ 2	☐ 3	☐ 4	☐ 5
4. When I feel sad, I feel like eating.	☐ 1	☐ 2	☐ 3	☐ 4	☐ 5
5. Eating helps me deal with negative emotions.	☐ 1	☐ 2	☐ 3	☐ 4	☐ 5

https://doi.org/10.3390/nu18101500

4. Emotional Awareness and Control in Eating

Compared to my usual behavior.	Strongly Disagree	Disagree	Neutral	Agree	Strongly Agree
1. I am aware that my emotions influence how much I eat.	☐ 1	☐ 2	☐ 3	☐ 4	☐ 5
2. I can distinguish between physical hunger and emotional hunger.	☐ 1	☐ 2	☐ 3	☐ 4	☐ 5
3. My appetite changes depending on my emotional state.	☐ 1	☐ 2	☐ 3	☐ 4	☐ 5
4. Certain emotions make me eat more or less than usual.	☐ 1	☐ 2	☐ 3	☐ 4	☐ 5
5. Understanding my emotions helps me regulate my eating behavior.	☐ 1	☐ 2	☐ 3	☐ 4	☐ 5

Table S2. Sociodemographic characteristics of the study sample (N = 411).

Characteristics	N	Percentage
**Gender**		
Male	95	23.1
Female	315	76.6
Missing	1	0.2
**Age**		
13-15	0	0.0
16-18	65	15.8
19-22	260	63.3
23-28	80	19.5
Missing	6	1.5
**Working or not**		
Exclusively Student	328	79.8
Working Student	83	20.2

Handling of Missing Sociodemographic Information. A small proportion of participants (1.5%) did not provide their exact age. Examination of the dataset indicated that these missing values were random, with no evidence of systematic non-response. All participants were recruited from the same higher-education context and therefore fall within the expected developmental range of Generation Z (late adolescence to young adulthood). Given the minimal proportion and random nature of the missing data, no imputation procedures were applied. These cases were retained in the analyses, as the absence of age information does not affect the representativeness of the sample nor the validity of the study's conclusions

https://doi.org/10.3390/nu18101500

Table S3. Mean values and standard deviations for all items assessing emotional influences on eating behavior. Values range from 1 (strongly disagree) to 5 (strongly agree). Response counts vary slightly across items (N = 408–411).

Question Code		Mean Value *	Standard Deviation
	**Emotional Undereating**		
Q1.1	I eat less when I am irritated	3.18	1.11
Q1.2	I eat less when I feel worried	3.27	1.32
Q1.3	I eat less when I feel anxious	3.20	1.41
Q1.4	I eat less when I feel sad	3.10	1.36
Q1.5	I eat less when I am angry	3.10	1.11
	**Reduced Enjoyment of Food under Emotional Distress**		
Q2.1	When I experience strong emotions, I have difficulty enjoying food	3.56	1.06
Q2.2	Emotional tension reduces my enjoyment of food	3.09	1.92
Q2.3	When I am emotionally upset, food tastes less pleasant to me	3.52	1.01
Q2.4	Negative emotions make me lose interest in food	3.22	1.20
Q2.5	When I feel emotionally pressured, I eat without pleasure	3.53	1.01
	**Emotional Eating/ Emotional Overeating**		
Q3.1	I feel a desire to eat when I am stressed	2.98	1.13
Q3.2	When I feel tense, I have a strong urge to eat	2.88	1.21
Q3.3	I use food as a way to cope with difficult emotions	2.61	1.19
Q3.4	When I feel sad, I feel like eating	2.98	1.20
Q3.5	Eating helps me deal with negative emotions	2.87	1.18
	**Emotional Awareness and Control in Eating**		
Q4.1	I am aware that my emotions influence how much I eat	3.70	0.93
Q4.2	I can distinguish between physical hunger and emotional hunger	3.67	1.04
Q4.3	My appetite changes depending on my emotional state	4.06	0.83
Q4.4	Certain emotions make me eat more or less than usual	3.95	0.92
Q4.5	Understanding my emotions helps me regulate me eating behavior	3.42	0.92

* Note: Values range from one to five.

https://doi.org/10.3390/nu18101500

Table S4. Exploratory factor analysis results for the four-factor model of post-COVID eating behavior (N = 411). Extraction method: PCA; Rotation: Varimax. Note. F1–F4 correspond to the latent factors extracted through exploratory factor analysis: F1 = Emotional Overeating (EO), F2 = Reduced Enjoyment (RE), F3 = Emotional Awareness (EA), and F4 = Emotional Undereating (EU). Communalities (h^2) indicate the proportion of variance in each item explained by the retained factors.

**Item**	**F1 (EO)**	**F2 (RE)**	**F3 (EA)**	**F4 (EU)**	**h^2**
Emotional Undereating (EU)					
1.I eat less when I feel irritated	-	-	-	0.851	0.783
2.I eat less when I feel worried	−0.620	-	-	-	0.580
3.I eat less when I feel anxious	−0.645	-	-	-	0.566
4.I eat less when I feel sad	−0.471	-	-	-	0.475
5.I eat less when I feel angry	-	-	-	0.847	0.751
Reduced Enjoyment (RE)					
1.When I experience strong emotions, I have difficulty enjoying food	-	0.823	-	-	0.721
2.Emotional tension reduces my enjoyment of food	-	0.831	-	-	0.697
3.When I am emotionally upset, food tastes less pleasant to me	-	0.784	-	-	0.670
4.Negative emotions make me lose interest in eating	−0.465	0.421	-	-	0.571
5.When I feel emotionally pressured, I eat without pleasure	-	0.721	-	-	0.596
Emotional Overeating (EO)					
1.I feel a desire to eat when I am stressed	0.788	-	-	-	0.633
2.When I feel tense, I have a strong urge to eat	0.798	-	-	-	0.691
3.I use food as a way to cope with difficult emotions	0.819	-	-	-	0.706
4.When I feel sad, I feel like eating	0.761	-	-	-	0.613
5.Eating helps me deal with negative emotions	0.754	-	-	-	0.573
Emotional Awareness (EA)					
1.I am aware that my emotions influence how much I eat	-	-	0.566	-	
2.I can distinguish between physical and emotional hunger	-	-	0.733	-	0.658
3.My appetite changes depending on my emotional state	-	-	0.788	-	0.658
4.Certain emotions make me eat more or less than usual	-	-	-	-	0.703
5. Understanding my emotions helps me regulate my eating behavior	-	-	-	0.440	0.192
Cronbach's α	0.877	0.825	0.487	0.793	
KMO = 0.90; Bartlett's $\chi^2(153) = 3450.12$, $p < 0.001$					
Total variance explained = 63.23%					

https://doi.org/10.3390/nu18101500

Table S5. Item-level descriptive statistics (Mean ± SD) for all 20 emotional-eating items, stratified by gender. Higher scores indicate stronger agreement with each statement (Likert scale 1–5). Missing data were minimal (0.2–0.7%).

Item	Men (n ≈ 94)	Women (n ≈ 315)
Q1.1	3.22 ± 1.12	3.17 ± 1.11
Q1.2	3.37 ± 1.30	3.24 ± 1.31
Q1.3	3.41 ± 1.37	3.13 ± 1.43
Q1.4	2.96 ± 1.29	3.14 ± 1.38
Q1.5	3.01 ± 1.14	3.13 ± 1.10
Q2.1	3.30 ± 1.17	3.64 ± 1.01
Q2.2	2.98 ± 1.18	3.12 ± 1.10
Q2.3	3.46 ± 1.03	3.54 ± 1.00
Q2.4	3.14 ± 1.23	3.25 ± 1.18
Q2.5	3.35 ± 1.12	3.58 ± 1.00
Q3.1	2.81 ± 1.24	3.03 ± 1.11
Q3.2	2.51 ± 1.15	2.99 ± 1.21
Q3.3	2.23 ± 1.07	2.72 ± 1.19
Q3.4	2.85 ± 1.17	3.02 ± 1.20
Q3.5	2.80 ± 1.14	2.90 ± 1.18
Q4.1	3.67 ± 0.96	3.71 ± 0.94
Q4.2	3.77 ± 0.97	3.64 ± 1.06
Q4.3	3.81 ± 0.85	4.14 ± 0.79
Q4.4	3.74 ± 0.94	4.02 ± 0.89
Q4.5	3.26 ± 0.96	3.47 ± 0.92

Table S6. Mean ± SD scores for the four emotional-eating subscales (Emotional Undereating, Reduced Enjoyment, Emotional Eating, Emotional Awareness) by gender. Subscale scores represent the average of the five corresponding items (Likert scale 1–5).

Subscale	Men (Mean ± SD)	Women (Mean ± SD)
Emotional Undereating (EU)	3.19 ± 1.25	3.16 ± 1.27
Reduced Enjoyment (RE)	3.25 ± 1.15	3.43 ± 1.06
Emotional Overeating (EO)	2.64 ± 1.16	2.93 ± 1.18
Emotional Awareness (EA)	3.65 ± 0.93	3.80 ± 0.92

https://doi.org/10.3390/nu18101500
